# Virtual patients as activities: exploring the research implications of an activity theoretical stance

**DOI:** 10.1007/s40037-014-0134-z

**Published:** 2014-08-01

**Authors:** Rachel H. Ellaway

**Affiliations:** Northern Ontario School of Medicine, 935 Ramsey Lake Road, Sudbury, ON P3E 2C6 Canada

**Keywords:** Virtual patient, Activity, Activity theory, Research, Scholarship, E-learning, Educational technology, Medical education

## Abstract

Virtual patients are computer-based simulators of patient encounters for the purposes of instruction, practice, and assessment. Although virtual patients have been around for some time they have yet to become part of mainstream medical education. A major reason for this would seem to be a lack of clarity as to what educational value virtual patients actually have. This paper argues that virtual patients should be seen as activities rather than artifacts and that activity theory can be used to generate different ways to frame scholarship in and around virtual patients. Drawing on the work of Leont’ev and Engeström this paper describes a range of perspectives based on the operations, actions, and objectives in and around virtual patients; the use of virtual patients to mediate activities; and the sociocultural context and the participants in virtual patient activities. This approach allows us to move beyond the ‘does or does not work’ discourse of much of the existing scholarship around virtual patients and, to an extent, around educational technologies as a whole. Activity perspectives, and activity theory in particular, offer new horizons for research and evaluation that address many of the limitations of intervention-based paradigms of inquiry.

## Introduction

Computer-based simulations of patient encounters have been a focus of interest in the medical education literature for some time [1, 2], often but not always under the label of ‘virtual patients’ [3, 4]. Some have sought to define or typify virtual patients and their many varieties [5]; others have explored how they should be designed and built [6, 7], and others have described how they can be used in particular curriculum contexts [8, 9]. A systematic review by Cook and Triola in 2009 concluded that ‘virtual patients should be designed and used to promote clinical reasoning skills’ [10]. An assumption more or less explicit in much of this work is that a virtual patient is an intervention, a catalyst that causally affords different or improved educational outcomes. This tends to equate the design of virtual patients to the design of the activities in which they will be used with most if not all of the activity encoded in the virtual patient itself.

The perspective that I explore in this paper is that educational value is realized through using tools and techniques in ways that are not defined solely by their design characteristics. I argue that we should therefore consider virtual patients in the broader context of the educational activities in which they are used, rather than as self-contained educational artifacts. This has implications for different dimensions of scholarship including original research, research synthesis, translational research, and the evaluation of teaching and learning processes [11]. Given the focus on activity as an organizing principle I draw on aspects of activity theory to create a model to guide and connect different approaches to scholarship in and around the use of virtual patients in educational activities.

## Activity and activity theory

The key concept in considering how educational artifacts (such as virtual patients) are used is the activity or activities constructed and performed around them. An activity consists of the specific actions and motivations of the participants engaged in a particular procedure in a particular place and time. While technical artifacts and the data they generate are concrete and easily observable, human activities are often less so, at least without some framework through which an activity can be understood. Activity theory developed as a body of thought focused on describing and understanding the complexities of human activity, and as such it provides a range of different perspectives and models that describe different aspects of what activity is and how it comes about. Different facets of activity theory can be applied to virtual patient activities to help us to understand what it is we should be paying attention to in and around them.

Leont’ev defined three levels within an activity; its overall *objectives*, the specific *actions* by which the broad objectives are to be addressed, and the stepwise *operations* that go to make up the actions [12]. Virtual patients have been described using this approach; the overall objectives of virtual patients (how they fit into a curriculum or programme of study), the actions constructed around the virtual patient (what teachers and learners do with and around them), and the operations needed to use the virtual patient (the clicks and key presses needed to make them run) [13]. In terms of scholarship these levels can equate to inquiry into why virtual patients are used, how they are used and how they work.

Engeström described activity systems where the subjects, objects and outcomes of an activity are bound to the mediating artifacts (tools and signs) that they employ and the sociocultural contexts in which they take place, in terms of the communities and individuals involved (both directly and indirectly), the rules by which these communities and individuals work, and the division of labour between them [14]. For example, a medical student (subject) may engage in a process of medical education (object) in order to qualify as a doctor (outcome). In doing so they engage with the curriculum, specific classes and sessions, as well as tools such as books, anatomical models, and simulators (mediating artifacts). This takes place in the context of a particular institution (community), various regulations (rules), and a distribution of responsibility and authority (division of labour).

Engeström also defined five principles of activity theory [15]: the design and orientation of activities around their object; the ways in which artifacts trigger actions; the relationships between specific actions and the activity as a whole; the ways in which contradictions and deviations change the activity; and the precedents and cultural norms the activity follows. Although there is much more to activity theory, and a full exposition is beyond the scope of this paper, these components have a particular relevance to the way we can understand virtual patient activities as they describe the structure of activities and activity systems, and the components within them.

## Redefining the scholarship of virtual patients

A previous paper explored how Leont’ev’s three levels of activity could be applied to virtual patients, noting that the nature of scholarly inquiry will change depending on the level under consideration [13]. This model can be developed by expanding on Leont’ev’s concepts and by incorporating Engeström’s concepts of activity systems, in particular the mediating role of artifacts and the role of context in defining and shaping activities.

Engeström states that artifacts function as mediating objects within activities [15]. An artifact may take many forms, from the concrete (books, buildings, software) to the more abstract (curricula, competences, systems). Although different artifacts mediate actions within an activity in different ways, the common concern is how and to what extent artifacts elicit or trigger these actions. We can therefore consider the mediating role of virtual patients within the activities in which they are used and the ways in which they trigger certain actions either at the individual level (such as scaffolding, pacing or sequencing a learning activity) or at the group level (such as gathering individuals with differing perspectives and abilities and helping them to move towards a common goal).

Engeström is also concerned with the ways in which activities are constructed and shaped, at least in part by the social and cultural contexts within which they are situated. Mediating artifacts are also defined and shaped by the social and cultural contexts in which they are used; something that other research into educational technologies confirms [16]. From the perspective of virtual patients we can consider both the influence of sociocultural context within which a virtual patient is used (in terms of its culture, community, and rules), and the influence of the backgrounds and personalities of the participants involved in the virtual patient activity and the roles they undertake as part of the activity.

**Fig. 1:**
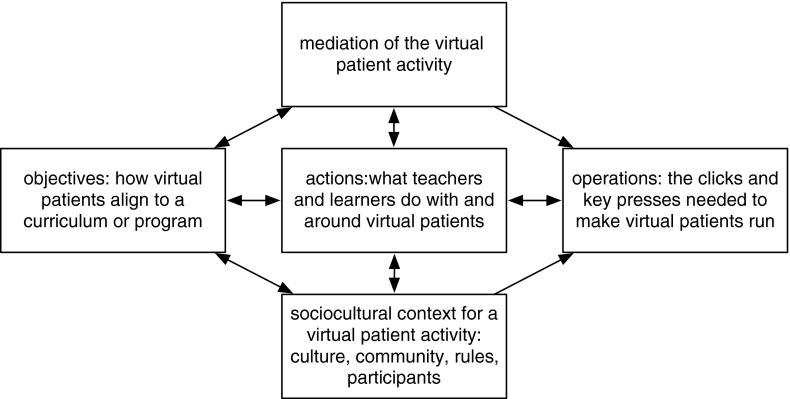
Five interconnected domains of virtual patient scholarship based on activity theoretical concepts derived from Leont’ev and Engeström

We can assimilate these five activity components (three from Leont’ev and two from Engeström) to create a model of the different approaches to scholarship that pertain to the efficacy and effectiveness of different virtual patient activities—see Fig. 1. Each of these five interconnected domains has different implications for research and evaluation.

### Virtual patient operations

This domain is concerned with how virtual patients work and how they are operated (the clicks and key presses needed to make them run). Virtual patients are almost exclusively screen-based and as such their operations are largely about operating a computer and the virtual patient software it runs, primarily by clicking on text and images and by typing on a keyboard. Research and evaluation in this domain would focus on these functional aspects of virtual patients. For instance, Mayer’s evidence-based multimedia principles have been used to guide the design of onscreen instructional materials [17] and could guide an exploration of virtual patient operations.

Concepts developed in the context of simulation, and in particular the design of simulators, can also provide opportunities for enquiry into virtual patient operations. For instance, Issenberg and McGaghie differentiate between two kinds of simulator, those that are: ‘static, permitting examination and manipulation by the student, but do not respond or provide feedback about what the learner is doing … [and those that are] interactive, responding in some way to student manipulations’ [18]. Virtual patients can also fall along this axis in terms of how much they can adapt and respond to learner decisions. Different kinds of activity have different implications for how virtual patients are used, which can in turn define their effectiveness. Another set of principles for guiding inquiry into the operations of virtual patients is Colvin-Clark’s work on ‘scenario-based learning’, which considers the importance of an activity’s adaptability to learner performance [19]. Research in and around virtual patient operations should bridge technical and educational discourses; for instance, considering what is possible (technical) and desirable (educational).

### Virtual patient actions

It is axiomatic to the thesis of this paper that the design features of a virtual patient are only realized through its use. Inquiry into virtual patient actions (what teachers and learners do with and around virtual patients) should therefore focus on how virtual patients could be used or are used. Although the great majority of the literature is explicitly or implicitly about virtual patient activities for self-directed independent study [10], there are many other ways in which virtual patients can be used both within and outside of classroom settings. For instance, virtual patients can be used in small groups with a focus on shared decision-making [20], they can be embedded in other activities such as problem-based learning (PBL) [8], and they can be used for assessment purposes [21].

Inquiry into virtual patient actions can also explore the bindings between a virtual patient and the activity in which it is used, particularly where it is not tightly bound into a one-to-one relationship. A single activity may involve the use of several different virtual patients [22], the same activity design may be rerun with different virtual patients addressing different topic areas [8], and a single virtual patient may be used in a range of different activities [16]. Inquiry can therefore focus on virtual patient activities in and of themselves without necessarily focusing on the specifics of the virtual patients they employ. Inquiry into virtual patient actions could also converge with general concepts of educational activity design and reuse, exploring issues such as what aspects of the design of a virtual patient may limit or enable its use in different actions or how actions can make use of different virtual patients [23].

### Virtual patient objectives

Inquiry into virtual patient objectives (how they fit into a curriculum or programme of study) should be concerned with the tactical and strategic reasons for using virtual patients, including the alignment between virtual patient activity objectives and outcomes, and the objectives and outcomes of the curriculum and programme of which the activity is a part, and the competencies learners are expected to develop through engaging in multiple virtual patient activities. This reflects a change in focus from single educational activities to educational methods (systematic interventions comprised of multiple related activities intended to serve broad educational purposes) [24]. Key questions from an objectives perspective should focus on the kinds of objectives, outcomes, and competencies that participation in virtual patient activities can afford, their efficacy and effectiveness in realizing them, and how they compare with the available alternatives.

### Virtual patients mediating activities

This domain is concerned with how activities can be (and are) mediated by using virtual patients, primarily focusing on the ways in which they elicit or trigger learning. For instance, virtual patients may scaffold activities by providing learners with structure, focus, agency, and clear indications of progress in attaining the intended goals [25]. Virtual patients may mediate an activity in similar ways to PBL cases by providing procedural structure in the form of triggers, pacing, and the activation of prior knowledge [26]. The exploration of the mediating role of virtual patients may also intersect with theories of simulation, in particular simplifying and abstracting real world situations, and affording learners’ agency that relates to future practice [27]. Inquiry into virtual patient activities could also be informed by social learning theories such as modelling desirable skills and behaviours, and allowing learners to explore the consequences of their actions [28].

Inquiry into the mediating role of virtual patients could be explored at the group or class levels as well as for individual learners. Indeed, existing research and evaluation practices favour studies that consider statistical changes in the collective performance of groups of learners [29–31]. Nevertheless, exploring the nature of virtual patients in mediating group activity could just as well explore the role they play in facilitating a particular group dynamic (such as problem-solving or planning a therapeutic response) or how they can respond to differences in participants’ knowledge, experience or motivation. Exploring the mediating role of virtual patients should also consider their combination with other mediating agents within an activity. For example, Edelbring et al. describe the impact of tutor mediation of virtual patient activities [32].

Virtual patient activities may also trigger or elicit unanticipated forms of learning or undesirable actions. For instance, a tutor or a virtual patient may diminish learning within a group setting by providing learners with too much guidance or direction, the mediating role of artifacts (virtual patients, tutors etc.) may need to be culturally responsive [33], learners may seek to ‘game’ a virtual patient to improve their marks [16], or the way the patient is represented in the virtual patient may send inappropriate messages to learners—a problem that has been identified in the context of PBL cases [34]. Inquiry into mediation in virtual patient activities should therefore also seek to identify and understand any unintended and emergent outcomes.

### Virtual patient activity contexts

It is not just mediating artifacts that can direct or change an activity; the contexts within which activities take place can also play an active role in shaping the ways in which they unfold. Inquiry into the role of context in virtual patient activities may work at a number of levels, from the individuals involved and their immediate activity setting through to the broader dynamics of a programme or school, or even to the influence of regional or national cuture and politics. For instance, the dynamics of virtual patient activities may change according to their participants’ training and cultural contexts [35].

An activity may change because of who its participants are or who they become during the activity. Factors include participants’ individual characteristics, their stage of training and the roles they take on within an activity [36]. Roles may be instructional (those from outside the activity, typically learner or teacher) or they may be task roles (those that are undertaken inside the activity). Although virtual patient activities can be designed so that learners do not take a particular role, professional learning activities can have greater impact if learners approach them in role [19], particularly if it is their target professional role.

Exploration of the broader sociocultural contexts for virtual patient activities is principally about uncovering and accounting for the cultural assumptions and norms that inform how virtual patient activities are constructed and used. Inquiry should focus on what is important, valued, or legitimate in and around virtual patient activities, and how this directs or impacts the practice of education and its outcomes.

For example, many schools have standard ways of conducting a patient consultation, often based on the ‘medical model’ of a linear sequence of history-taking, physical examinations, tests and investigations, diagnoses, and therapies and interventions, which is reflected in the format of a great many virtual patients [7], particularly it would seem by schools with more of a tertiary teaching hospital focus. Other schools and programmes may have different perspectives and needs. For instance, virtual patients for community programmes may need to be more about population health and the influence of different social determinants of health. Inquiry may consider other cultural variables, such as the acceptability of ambiguity in the case details or the instructional model, the acceptability of negative outcomes (such as unprofessional behaviour or the patient’s demise), or other emotional aspects of a virtual patient activity.

## In practice

There are many ways in which the approach and the framework I have set out could be used in practice. Recognizing this plurality, I provide the following as an illustration of these domains in action. These examples are based on a hypothetical study into a team-based learning activity that involves a class of second-year medical students alternating between short lectures as a whole group and small group virtual patient activities. The virtual patient component involves learners working in pairs to apply concepts and knowledge from the lecture to solve the problems presented by the virtual patient. Options for designing and conducting research into this virtual patient activity can be organized around the five domains set out in the previous section:

- *Operations:* one approach would be to take the virtual patient as a fixed entity, focusing inquiry on what learners have to do to negotiate the activities and how these operations impact their learning experiences and their outcomes. An alternative approach would be to take the intended operations as fixed to ask what aspects of virtual patient design could afford these desired operations. In both circumstances this could involve quantifying the effort, the resources, or the expertise required, or it could be about describing or explaining them.
- *Actions:* research oriented around this domain could involve exploring the dynamics of the small group work around the virtual patients, such as the discussion and debate amongst each pair as they move through different sub-activities. It could also involve looking at the dependencies and connections between the different virtual patient sub-activities and their contribution to the team-based learning session as a whole.
- *Objectives:* research drawing on this domain could consider the reasons why virtual patient activities were selected for the team-based learning session, including the objectives for each virtual patient sub-activity. Alternatively, it could focus on what objectives are possible or advisable for this kind of activity, and how they relate to the objectives for the team-based learning session and to the curriculum as a whole.
- *Mediating activities:* research from this domain could explore the use of sequencing, pacing, and triggers in virtual patient sub-activities, and the ways in which learning is (or is not) supported by these different components. It could also consider how the activity relates to the real-world equivalents of the cases or situations the virtual patient activities represent. Alternatively, research could investigate how learners learn (or do not learn) from participating in the virtual patient sub-activities, what mechanisms are involved in facilitating their learning, and what effect they have on the quality of the learning outcomes for one or more of the participating small groups.
- *Activity context:* research from this domain could consider the cultural assumptions and norms of the school and programme, and how they relate to the choice and design of the virtual patients and the virtual patient activities in which they are used. It could also involve analyzing the discourses and social dynamics within the virtual patients themselves, such as how the patient is represented or how author, teacher and learner attitudes and assumptions are realized in the conduct of different sub-activities. Alternatively, research could explore how different learners respond to undertaking different roles within a virtual patient activity. It could also consider how participants’ previous experience can change the experiences of participants or change the outcomes of different virtual patient activities.

These approaches should not necessarily be used independently. Indeed, real world inquiry would be expected to draw on several domains to be able to say something meaningful about a particular situation. For instance, a study may relate activity design to objectives and outcomes while another may be based on relating participant roles to the actions they perform and the efficacy of different intersections between the two. Furthermore, research that draws on these domains (individually or in combination) may simply explore ‘what worked in this situation?’ or it could take a more design-based approach by asking ‘how should we design or change this situation to optimize its efficacy and efficiency?’

## Discussion

It would be unusual for an experienced medical educator to mistake a PBL case for a PBL activity, so it may be a particular quirk of virtual patients that they are so often mistaken for the activities in which they are used. However, it is arguable that this artifact-activity tension applies to many other educational technologies, such as social media, portfolios, smartphones, and virtual worlds. A framework derived from concepts in activity theory may therefore have a wider applicability, although more work would be needed to explore this.

I have selected certain aspects of activity theory to construct this framework based on their utility in describing different aspects of virtual patient activities. Activity theory is much larger and complex than I have been able to represent in this paper and it could be further explored to inform the scholarship of virtual patients. For instance, Engeström’s more recent work has explored the concept of ‘knots’, the ways in which loosely connected activities are dynamically linked and unlinked [15]. This perspective could be used to explore interactions and interdependencies between virtual patient, problem-based learning, and simulation activities. Activity theory is not the only theoretical stance that can relate to virtual patient activities. As an example, realist methods explore and explain different mechanisms that achieve certain outcomes in certain contexts [37] and could be used to explain ‘how’ virtual patients work as a precursor to, or in parallel to, studies that explore ‘if’ they work. Another field that has the potential to inform this area is design-based research [38] as virtual patients and the activities that make use of them have an intrinsic design component.

This paper is presented in the context of calls for more deliberate and robust use of theory in medical education research [39, 40], with a particular focus on the exploration and use of socio-cultural learning theories [41, 42], as a way of establishing what is and is not ‘good’ evidence to inform medical education scholarship [43]. Using multiple lenses to research and evaluate educational technologies can yield valuable insights that a single approach cannot access [44], and as such, a pluralistic approach to exploring and undertaking scholarship in and around virtual patients is, I argue, a critical response to those who simply ask whether or not virtual patients work. In making this assertion we should be clear that the framework I have set out is intended to help to expand and diversify the way we approach research and evaluation in and around virtual patients. It is also intended to make the relationships and dependencies between virtual patients and the activities in which they are used more explicit and tractable to scholarly inquiry. Although I have tended to focus on primary research, I hope that this framework will inform different kinds of scholarship including systematic and thematic reviews, translational research, and the systematic evaluation of teaching and learning systems.

## Conclusions

We clearly need to move beyond the ‘works’/’not works’ discourse of much of the existing scholarship around virtual patients, and for that matter, around educational technologies as a whole. I have argued that we should not simply ask whether a virtual patient works any more than we would ask whether a PBL case works. It is the virtual patient activity that is the educational intervention or mechanism and as such it should be the main focus of our attention rather than the technological artifacts that are used within it. There is an emerging discourse around activity within the virtual patient literature [13, 20, 45] and this paper is presented in that context. Activity perspectives, and activity theory in particular, offer new horizons for research and evaluation that have the potential to address many of the limitations of an intervention-based paradigm. By using them we might at last be able to demonstrate and make use of the real value (be it high or low) of using virtual patients in medical education.

## Essentials

1. Virtual patients are computer-based simulations of patient encounters but it is unclear what educational value they have.
2. This paper argues that it is the use of virtual patients and the ways that they are used that confers educational value. This use is encapsulated in the concept of ‘virtual patient activities’.
3. Activity theory can be used to generate different approaches to frame scholarship in and around virtual patient activities.
4. Approaches to virtual patient activity scholarship include exploring the operations, actions, and objectives in and around virtual patients; the role of virtual patients in mediating activities; and the sociocultural context and the participants in virtual patient activities.
5. An activity lens may generalize to educational technology in general but more work is required to explore this approach.
